# Predictive value of prognostic nutritional index (PNI) in recurrent or unresectable hepatocellular carcinoma received anti-PD1 therapy

**DOI:** 10.1186/s12885-023-11166-w

**Published:** 2023-08-23

**Authors:** Xindan Kang, Jing Wang, Xue Kang, Li Bai

**Affiliations:** 1 Department of Respiratory and Critical Care Medicine, The Second Medical Center & National Clinical Research Center for Geriatric Diseases, Chinese People’s Liberation Army General Hospital, Beijing, 100089 China; 2 Department of Oncology, The First Medical Center, Chinese People’s Liberation Army General Hospital, Beijing, 100036 China; 3 Department of General Medicine, The First Medical Center, Chinese People’s Liberation Army General Hospital, Beijing, 100036 China

**Keywords:** Immune checkpoint inhibitors, Unresectable hepatocellular carcinoma, PNI, Anti-PD1

## Abstract

**Background:**

Clinical trials have shown that anti-PD1 therapy, either as a monotherapy or in combination, is effective and well-tolerated in patients with recurrent or unresectable hepatocellular carcinoma (HCC). In this study, we aimed to investigate the prognostic value of immune-nutritional biomarkers in measuring the effects of anti-PD1 therapy in these patients.

**Methods:**

We enrolled and followed up with 85 patients diagnosed with advanced HCC who underwent anti-PD1 therapy at the First Medical Centre of Chinese People’s Liberation Army (PLA) General Hospital between January 2016 and January 2021. The retrospective analysis aimed to determine whether immune-nutritional biomarkers could serve as promising prognostic indices in these patients.

**Results:**

In this retrospective study, patients in the PNI-high group showed a better progression-free survival (PFS) compared to those in the PNI-low group (9.5 months vs. 4.2 months, P = 0.039). Similarly, the median overall survival (OS) was longer in the PNI-high group (23.9 months, 95%CI 17.45–30.35) than in the PNI-low group (11.7 months, 95%CI 9.27–14.13) (P = 0.002). These results were consistent with sub-analyses of the anti-PD1 therapy. Furthermore, both univariate and multivariate analyses indicated that a higher pre-treatment PNI ( > = 44.91) was a significant predictive factor for favorable outcomes in this patient cohort (HR = 0.411, P = 0.023).

**Conclusion:**

Our study suggests that pre-treatment PNI is a critical predictive factor in patients with recurrent or unresectable HCC undergoing anti-PD1 therapy.

## Introduction

Primary liver cancer is one of the most common malignant tumors affecting the digestive tract and is currently the third leading cause of cancer-related death worldwide, according to a 2020 report by the International Agency for Research on Cancer [1]. Hepatocellular carcinoma (HCC) is the most prevalent pathological pattern of primary malignancy in the liver, accounting for approximately 85-90% of cases. Surgical resection, liver transplantation, and locoregional therapy are curative local modalities for early-stage HCC patients [2, 3]. However, the high recurrence rate after radical operation remains a challenge. Furthermore, HCC’s asymptomatic pre-clinical stage and limited systemic treatment options contribute to poor prognosis and high mortality. As a highly invasive and refractory cancer, HCC poses a serious threat to people’s health and life in China [1, 4, 5]. Before 2017, sorafenib was the only treatment available for patients with unresectable HCC, but its effectiveness was extremely limited [6]. Since then, lenvatinib has been approved by the Food and Drug Administration as an alternative first-line treatment for patients with unresectable HCC [7]. Several antiangiogenic agents (regorafenib, ramucirumab, apatinib, cabozantinib, etc.) are also considered optimal second-line treatments after sorafenib treatment [8–10]. In addition to targeted therapy, immunotherapy using immune checkpoint inhibitors (ICIs) is a novel approach to treating tumors and has shown promising results in increasing survival rates and improving prognosis. Therefore, developing predictive biomarkers to screen patients who could benefit from anti-programmed cell death protein 1 (anti-PD1) therapy is of utmost importance.

Since 2017, monotherapy with nivolumab, pembrolizumab, and camrelizumab has offered a new approach for second-line treatment of unresectable HCC [11–13]. Additionally, combining anti-PD1 with various antiangiogenic agents or nivolumab (1 mg/kg) and ipilimumab (3 mg/kg) has shown to be more effective than immune monotherapy [14]. The objective response rate (ORR) for anti-PD1 monotherapy in several solid tumors is approximately 15-20%, which is much lower than that in highly immunogenic unresectable melanoma and Hodgkin’s disease [15]. However, HCC, being an inflammation-associated immune cancer, may evade the immune system’s surveillance by targeting immune checkpoints. Therefore, ICIs are considered a breakthrough in the therapeutic regimen of advanced HCC. Available research has shown that combination therapy with immunotherapy and antiangiogenic therapy is superior to either module alone [16]. Additionally, finding a promising predictive prognostic biomarker in unresectable HCC patients who receive immunotherapy remains an open question.

Previous studies have demonstrated that chronic inflammation is one of the dominant characteristics in tumorigenesis and significantly impacts prognosis [17]. Aspirin, a nonsteroidal anti-inflammatory drug (NSAID), has been found to provide better disease-free survival and overall survival when compared to non-NSAID users [17]. Immunoinflammatory biomarkers, such as C-reactive protein (CRP), prognostic nutritional index (PNI), platelet-to-lymphocyte ratio (PLR), neutrophil-to-lymphocyte ratio (NLR), and the systemic immune-inflammation index (SII), have shown promising predictive value for prognosis in hepatocellular carcinoma (HCC) patients [18–20]. Compared to conventional methods like tumor-node-metastasis (TNM) stage and vascular invasion, these biomarkers may provide more convenient prognostic parameters for inoperable HCC patients. Though programmed death-ligand 1 (PD-L1), microsatellite instability (MSI), and tumor mutational burden (TMB) are potential biomarkers that can select patients in solid tumors like colorectal cancer and non-small cell lung cancer (NSCLC) who are likely to benefit from immune checkpoint inhibitor (ICI) therapy. However, PD-L1 expression greater than 1% in tumor cells only occurs in approximately 20% of HCC patients, and the prevalence of MSI in HCC is only about 0.8-3% [21, 22]. Moreover, there was no significant relationship between TMB and response rates in 755 patients with unresectable HCC [23]. Therefore, non-invasive methods that rely on inflammation-associated indicators derived from peripheral blood are urgently needed, especially considering the availability and adequacy of tumor tissue.

Therefore, the objective of this retrospective observational study is to provide additional clinical evidence regarding the use of ICIs as a treatment option for patients with unresectable HCC.

## Materials and methods

### Study population and design

Patients with recurrent and unresectable HCC who received anti-PD1 therapy were screened between January 2016 and January 2021 at the First Medical Centre of Chinese PLA General Hospital. Demographic and clinical characteristics were collected independently by two physicians from medical records at baseline prior to immunotherapy, including age, gender, disease stage, pathological subtypes, history of smoking, drinking, allergies, and prior treatments. Eastern Cooperative Oncology Group performance status (ECOG-PS), Barcelona Clinic Liver Cancer (BCLC) stage, Child-Pugh Score, prior systemic and locoregional therapies, and follow-up information were also collected. Peripheral blood tests, including complete blood count, blood biochemical indices, tumor markers, and indicators of HBV infection, were routinely performed before ICIs therapy. Personal information was kept confidential in this retrospective study, which was approved by the Ethics Committee of the First Medical Centre of Chinese PLA General Hospital.

### Inclusion and exclusion criteria

Inclusion criteria:

1. Patients over the age of 18 with unresectable HCC and recurrent HCC.
2. Patients received more than one dose of Anti-PD1 therapy and at least one therapeutic evaluation.
3. The original histological type was HCC confirmed by pathology, rather than clinical diagnosis (radiographic findings or serological diagnosis, for instance).
4. Peripheral blood test results were performed within one weeks before initiation treatment.
5. The child-pugh (A or B) and BCLC classification of HCC (B or C).
6. Unresectable and recurrent HCC based on Milan Criteria and multidisciplinary team (including medical oncologists, surgeons, radiologists, radiation oncologist, etc.).

Exclusion criteria:

1. Patients with a history of other malignant cancers.
2. Patients with autoimmune diseases and infectious diseases that required therapy.
3. Patients previously treated with any biological immunization therapy targeting T cell like cytokine-induced killer cells, mature dendritic cells and dendritic cells-natural killer T cells, etc.
4. Patients without insufficient laboratory tests data or incomplete clinical data.

### Definitions

#### Inflammatory indicators

1. Prognostic nutritional index (PNI) = peripheral blood albumin level (g/L) + 5* absolute value of peripheral lymphocytes (10^9/L);
2. Neutrophil to lymphocyte ratio (NLR) = absolute neutrophil count in peripheral blood (10^9/L)/absolute lymphocyte count in peripheral blood (10^9/L);
3. Platelet to lymphocyte ratio (PLR) = absolute platelet count of peripheral blood (10^9/L)/absolute lymphocyte count of peripheral blood (10^9/L);
4. Systemic immune-inflammation index (SII) = absolute neutrophil count in peripheral blood (10^9/L) *absolute platelet count in peripheral blood (10^9/L)/absolute lymphocyte count in peripheral blood (10^9/L);

Using the area under the receiver-operating characteristic curve (ROC) to identified the cut-off value. According to the cut-off value, the patients were divided into high and low groups.

#### Evaluation index

Baseline data for all patients before anti-PD1 therapy included multiphase dynamic computed tomography (CT) scanning of the thorax and abdomen, or abdominal magnetic resonance imaging (MRI). Radiological evaluations of the liver were conducted every 8–12 weeks. The eighth edition of the American Joint Committee on Cancer TNM staging system and BCLC scores were used to classify the HCC stage [24]. The therapeutic effect was evaluated using the Response Evaluation Criteria in Solid Tumors (RECIST 1.1), which includes complete response (CR), partial response (PR), stable disease (SD), and progressive disease (PD). Furthermore, the proportion of patients with at least CR or PR as the best overall response was defined as the objective response rate (ORR), while the disease control rate (DCR) was calculated as the sum of ORR with the addition of SD.

#### Follow up

Progression-free survival (PFS) was defined as the time from the first day of initial anti-PD1 treatment to the day of disease progression by radiological findings or death from any cause, whichever occurred first. For patients without tumor recurrence, the time was calculated up to the end of the last follow-up. Overall survival (OS) was defined as the time from the initial anti-PD1 treatment to the date of the last follow-up or the time of death regardless of cause. For patients who missed a follow-up visit during the study period, PFS and OS were calculated up to the day of the last follow-up visit. This retrospective cohort study of patients was routinely followed up until August 2022.

## Statistical analysis

Demographic characteristics were presented using frequencies and percentages for categorical variables and medians and ranges for quantitative variables. Survival curves for progression-free survival (PFS) and overall survival (OS) were estimated using the Kaplan-Meier method. Receiver operating characteristic (ROC) curves of inflammatory indicators for diagnosing one-and-a-half-year survival were plotted, and the optimal cut-off values were obtained using the Youden index. Furthermore, univariate and multivariate analyses via Cox’s proportional hazard regression can be used to determine risk factors. Variables with P < 0.2 in the univariate analysis were included in the multivariate analysis. All statistical tests were two-sided, and P-values < 0.05 were considered statistically significant.

## Results

### Baseline characteristics

**Fig. 1:**
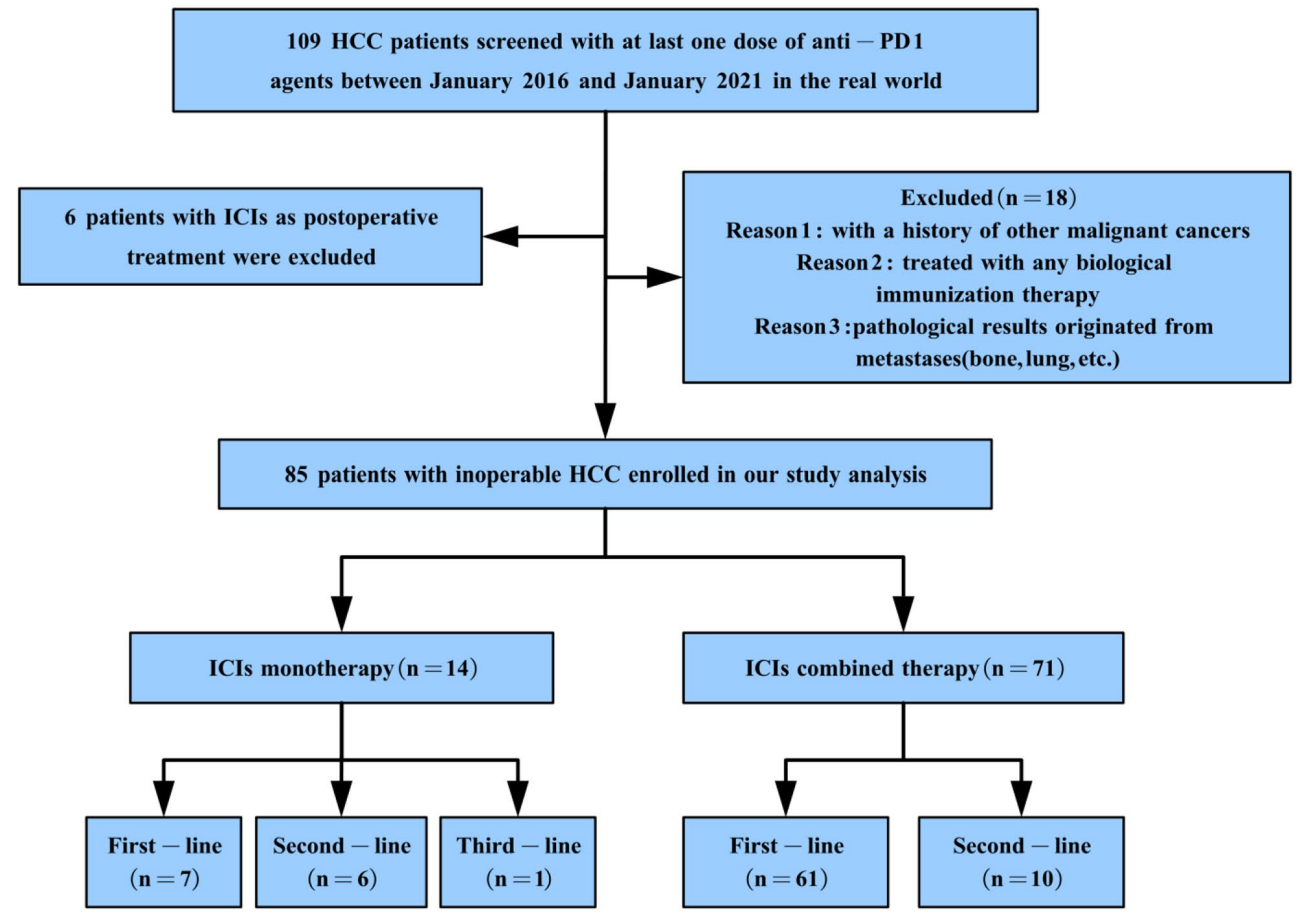
The data flow diagram of our study

A total of 109 HCC patients receiving ICIs between January 2016 and January 2021 were screened for inclusion in this study. Adjuvant immunotherapy was given postoperatively to 6 patients who were excluded from the study. Finally, 85 patients met the inclusion and exclusion criteria and were included in the result analysis, with 71 in the anti-PD1 combination group and 14 in the anti-PD1 monotherapy group (see Fig. 1). Table 1 reports the baseline demographics and clinical characteristics of unresectable HCC patients. The advanced HCC patients receiving immunotherapy had an average age of 52.1 years. Among the 85 subjects (74 males and 11 females), the main etiology of liver disease was hepatitis B virus (88.2%). The majority of patients with HCC had liver cirrhosis (71.8%) and well ECOG performance status (96.5%). The percentages of smokers, drinkers, and patients with allergies were 51.8%, 61.2%, and 12.9%, respectively. Liver function was determined using the Child-Pugh score, with 89.4% of patients having concomitant Child-Pugh class A and 10.6% of patients having Child-Pugh class B liver function. According to the BCLC classification of HCC, 18.8% and 81.2% of patients had B and C stages of the disease, respectively, and 45.9% had extrahepatic metastasis, 30.6% had vascular invasion, and 38.8% had lymph node metastasis, indicating that the patients were unresectable. At the final follow-up, 20 patients were still alive, and 5 cases were withdrawn. All cases were followed up for a range of 2.4 months to 65.4 months.

**Table 1:** Demographics and clinical characteristics of unresectable HCC patients

Clinical Factor	All Patients (n = 85)	Anti-PD1 combination therapy (n = 71)	Anti-PD1 monotherapy (n = 14)	P-value		
	No.(%)	No.	%	No.	%	
Median age (range)	54 ± 10.53 (24–77)	54 ± 11.04 (24–77)	52 ± 7.65 (44–71)			
Age						0.741
>=55 years < 55 years	40(47.1) 45(52.9)	34 37	47.9 52.1	6 8	42.9 57.1	
Gender						0.409
Male Female	74 (87.1) 11 (12.9)	63 8	88.7 11.3	11 3	78.6 21.4	
ECOG performance status						0.440
0 >=1	82 (96.5) 3 (3.5)	68 3	95.8 4.2	14 0	100.0 0.0	
Smoking history						0.890
Current or former Never	44 (51.8) 41 (48.2)	37 34	52.1 47.9	7 7	50.0 50.0	
Drinking history						0.158
Current or former Never	52 (61.2) 33 (38.8)	46 25	64.8 35.2	6 8	42.9 57.1	
Allergic history						0.410
Yes No	11 (12.9) 74 (87.1)	10 61	14.1 85.9	1 13	7.1 92.9	
HBV infection						0.171
Negative Positive	10 (11.8) 75 (88.2)	7 64	9.9 90.1	3 11	21.4 78.6	
Portal vein thrombosis						0.038
Yes No	26 (30.6) 59 (69.4)	25 46	35.2 64.8	1 13	7.1 92.9	
Extrahepatic metastasis						0.811
Yes No	39 (45.9) 46 (54.1)	33 38	46.5 53.5	6 8	42.9 57.1	
Perihepatic lymph node metastasis						0.800
Yes No	33 (38.8) 52 (61.2)	28 43	39.4 60.6	5 9	35.7 46.3	
Child-Pugh class						0.674
A B	76 (89.4) 9 (10.6)	64 7	90.1 9.9	12 2	85.7 14.3	
BCLC score						0.393
B C	16 (18.8) 69(81.2)	12 59	16.9 83.1	4 10	28.6 71.4	
Tumor number						0.712
Single Multiple	15(17.6) 70(82.4)	13 58	18.3 81.7	2 12	14.3 85.7	
Tumor size						0.551
>=5 < 5	49(57.6) 36(42.4)	42 29	59.2 40.8	7 7	50.0 50.0	
Liver cirrhosis						0.539
Absence Presence	24(28.2) 61(71.8)	19 52	26.8 73.2	5 9	35.7 46.3	
Ascites						0.502
Absence Presence	54(63.5) 31(36.5)	44 27	62.0 38.0	10 4	71.4 28.6	
Operation history						0.566
Yes no	55(64.7) 30(35.3)	45 26	63.4 36.6	10 4	71.4 28.6	
TACE simultaneously						0.605
Yes No	25 (29.4) 60(70.6)	20 51	28.2 71.8	5 9	35.7 46.3	
Treatment lines of immunotherapy						0.002
1 >=2	68(80.0) 17(20.0)	61 10	85.9 14.1	7 7	50.0 50.0	
Prior systemic therapy						0.002
No Yes Sorafenib Lenvatinib Others	68(80.0) 17(20.0) 10(58.8) 4(23.5) 3(17.7)	61 10 6 3 1	85.9 14.1 60.0 30.0 10.0	7 7 4 1 2	50.0 50.0 57.1 14.3 28.6	
AFP						0.783
>=20 < 20	48(56.5) 37(43.5)	43 28	60.6 39.4	5 9	35.7 46.3	
PNI						0.138
>=44.91 < 44.91	49(57.6) 36(42.4)	44 27	62.0 38.0	5 9	35.7 46.3	
Therapeutic effect evaluation, n (%)						
CR PR SD PD	1(1.2) 33(38.8) 32(37.6) 19(22.4)	1 31 27 12	1.4 43.7 38.0 16.9	0 2 5 7	0 14.3 35.7 50.0	
ORR	34(40.0)	32	45.1	2	14.3	0.012
DCR	66(77.6)	59	83.1	7	50.0	0.037

### Therapy characteristics and optimal cut-off values of systemic inflammatory response biomarkers

In this retrospective study, over 60% of the individuals underwent surgical intervention, with three cases receiving a liver transplant and nine unresectable HCC patients undergoing anti-PD1 combination therapy as first-line treatment, presenting a new opportunity to undergo an operation. Additionally, one in five patients (17/85) received at least one palliative systemic therapy before starting ICIs, while four out of five patients received immunotherapy as their first-line therapy. Among the first-line therapeutic agents, 10.3% (7/68) of patients received anti-PD1 monotherapy, while the rest received anti-PD1 combination therapy.

**Fig. 2:**
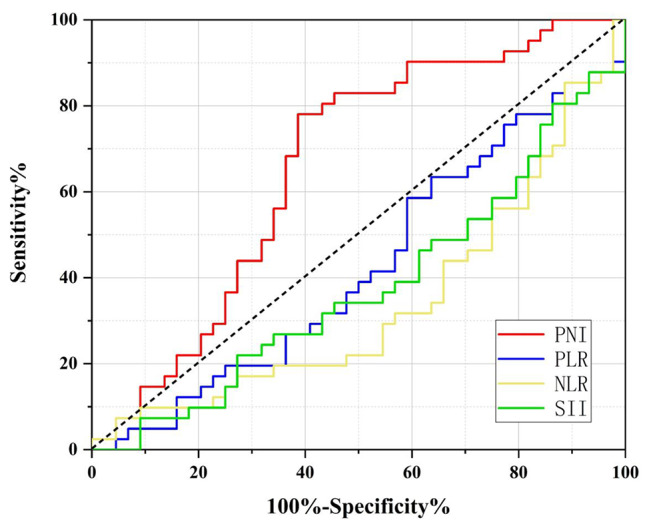
A predictive OS ability of the PNI was compared with other inflammatory indicators (PLR, SII and NLR) by ROC curves

The predictive ability of PNI was evaluated by comparing it with other inflammatory biomarkers (PLR, NLR, and SII) using a ROC curve analysis. The results showed that PNI had a higher discrimination ability for overall survival (OS) compared to other inflammatory indicators, with an area under the curve (AUC) of 0.613. Using the ROC curve and the Youden index, the recommended baseline PNI cutoff was determined to be 44.91. The corresponding sensitivity and specificity of the PNI cutoff values were found to be 78.9% and 59.6%, respectively, as shown in Fig. 2. Furthermore, in the follow-up study, we were able to confirm the impact of PNI on survival outcomes.

### Efficacy and survival analysis

**Fig. 3:**
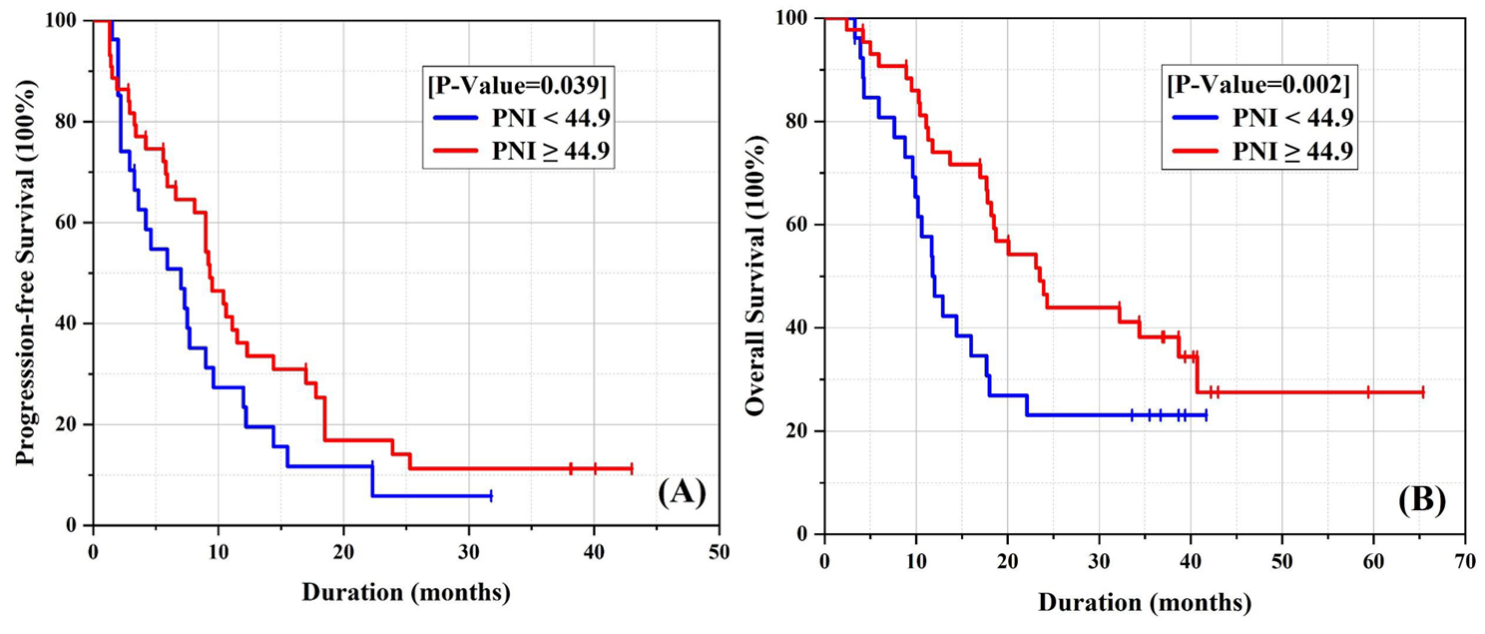
Kaplan-Meier estimates of PFS (**A**) and OS (**B**) in 85 advanced patients receiving ICIs. (**A**) PFS according to PNI (high- vs. low-group). (**B**) OS according to PNI (high- vs. low-group)

**Fig. 4:**
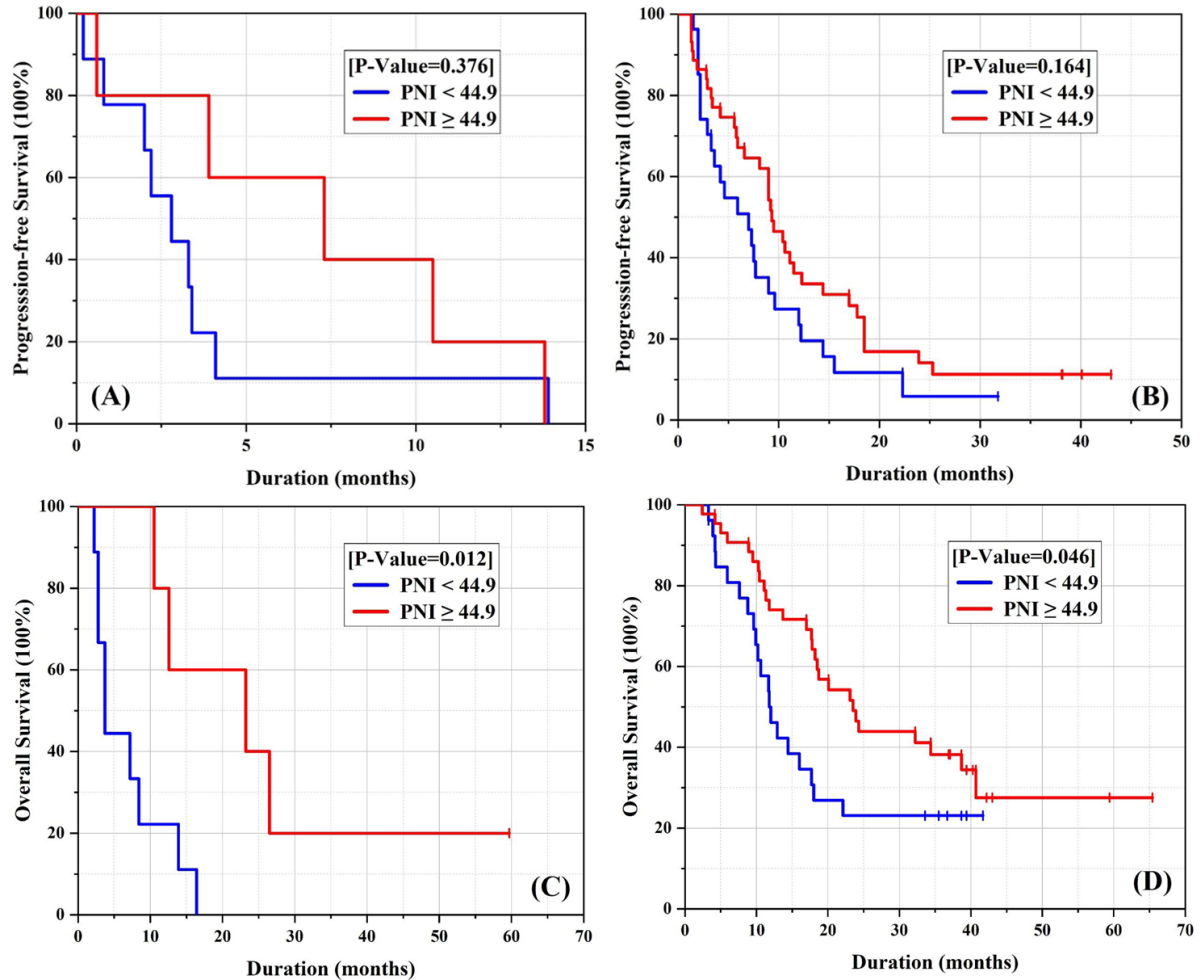
Subgroup analysis of PFS and OS. For those patients receiving anti-PD1 monotherapy, the median PFS was 2.80 months (95%CI 1.05–4.56) and median OS was 3.7 months (95%CI 2.39–5.02) with PNI-low group, while the median PFS was 7.3 months (95%CI 0.00-14.60) and median OS was 23.20 months (95%CI 0.44–45.96) with PNI-high group (Fig. 4A and C). Likewise, in combination therapy, the median PFS was 7.3 months (95%CI 3.75–10.85) and median OS (12.00 months, 95%CI 8.63–15.37) with PNI-low group was also shorter than the median PFS was 9.5 months (95%CI 7.59–11.41) and median OS (23.90 months, 95%CI 16.64–31.16) with PNI-high group

According to the radiological RECISTv1.1 criterion, a remarkable overall response rate (ORR) of 38.8% (19/49) and disease control rate (DCR) of 83.7% (41/49) were observed, including one patient with complete response (CR) and 18 patients with partial response (PR) in the PNI-high group. In addition, stable disease was documented in 22 patients (44.9%) and 8 patients (16.3%) showed progressive disease as the best response to ICIs, as shown in Table 2. The PNI-high group demonstrated a higher DCR compared to the PNI-low group (83.7% vs. 69.5%, P = 0.137) (Table 2). For these advanced HCC patients who received ICIs therapy, our preliminary results showed that the median progression-free survival (PFS) was 9.5 months (95%CI 7.60-11.41) and the median overall survival (OS) was 23.9 months (95%CI 17.45–30.35) in the PNI-high group. However, the median PFS was 4.2 months (95% CI 0.94–7.46) and the median OS was 11.7 months (95% CI 9.27–14.13) in the PNI-low group, as shown in Fig. 3. Similar results were obtained in the subgroup analysis, as shown in Fig. 4.

**Table 2:** The tumor response in each treatment group

Best overall response, n (%)	the PNI high group ( > = 44.91, n = 49)	the PNI-low group (< 44.91, n = 36)	P-value
Complete response, n (%)	1 (2.1)	0 (0.0)	
Partial response, n (%)	18 (36.7)	15 (41.7)	
Stable disease, n (%)	22 (44.9)	10 (27.8)	
Progressive disease, n (%)	8 (16.3)	11 (30.5)	
Objective response rate, n (%)	19 (38.8)	15 (41.7)	0.792
Disease control rate, n (%)	41 (83.7)	25 (69.5)	0.137

### The patient in the PNI-high group achieving complete remission

In the PNI-high group (with PNI values greater than or equal to 44.91), one patient achieved complete remission. This patient had portal vein tumor embolus and was treated with surgical intervention. However, AFP levels continued to rise, indicating recurrence within one year. Following prophylactic treatment with TACE and sorafenib therapy, recurrence was detected in the form of lung metastasis rather than in situ. Currently, the patient has achieved complete remission after receiving 40 cycles of anti-PD1 and lenvatinib, and their condition remains stable. There were no severe adverse reactions reported, only mild stomach upset.

### Univariate and multivariate Cox regression analyses for the unresectable HCC patients

According to the univariate analysis, it identified that Child-Pugh class (HR = 1.932; 95%CI 0.914–4.082; P = 0.084), Portal vein thrombosis (HR = 0.622; 95% CI 0.371–1.045; P = 0.073), Perihepatic lymph node metastasis (HR = 0.726; 95% CI 0.451–1.166; P = 0.185), BCLC score (HR = 1.678; 95%CI 0.926–3.040; P = 0.088), Operation History (HR = 0.691; 95% CI 0.425–1.125; P = 0.138), Treatment lines of immunotherapy (HR = 1.512; 95% CI 0.839–2.726; P = 0.169), PNI (HR = 0.617; 95% CI 0.388–0.983; P = 0.042), Therapeutic regimen (HR = 0.387; 95% CI 0.212–0.708; P = 0.002) and therapeutic effect evaluation (HR = 3.810; 95% CI 2.192–6.622; P < 0.001) were markedly correlated with PFS in unresectable HCC patients who underwent anti-PD1 therapy (Table 3). On multivariate analysis, a lower PNI (< 44.91) and worse response rate (SD + PD) showed a trend toward reduce PFS (P = 0.003 and P < 0.001). Most surprisingly, even in portal vein thrombosis (P = 0.042).

**Table 3:** Univariate and multivariate Cox regression analyses for PFS in unresectable HCC patients

	Progression-Free Survival Univariate			Progression-Free Survival Multivariate		
Variables	HR	95%CI	P-value	HR	95%CI	P-value
Age						
< 55 years	1					
>=55 years	0.808	0.509–1.284	0.367			
Gender						
Feale	1					
Male	1.015	0.503–2.050	0.967			
ECOG-PS						
0	1					
>=1	0.987	0.309–3.151	0.982			
Smoking history						
Never	1					
Current or former	1.094	0.690–1.736	0.702			
Drinking history						
Never	1					
Current or former	0.818	0.509–1.312	0.404			
Allergic history						
No	1					
Yes	0.644	0.319–1.303	0.221			
HBV infection						
Negative	1					
Positive	1.036	0.529–2.028	0.918			
Child-Pugh class						
A	1			1		
B	1.932	0.914–4.082	0.084	1.427	0.579–3.518	0.440
Portal vein thrombosis						
No	1			1		
Yes	0.622	0.371–1.045	0.073	0.536	0.294–0.979	0.042
Extrahepatic metastasis						
No	1					
Yes	1.242	0.782–1.972	0.358			
Perihepatic lymph node metastasis						
No	1			1		
Yes	0.726	0.451–1.166	0.185	0.793	0.458–1.373	0.407
BCLC score						
B	1			1		
C	1.678	0.926–3.040	0.088	0.814	0.414–1.598	0.549
Tumor number						
Single	1					
Multiple	1.155	0.621–2.147	0.650			
Tumor size						
=<5	1					
> 5	1.006	0.632-1.600	0.981			
Liver cirrhosis						
Absence	1					
Presence	0.818	0.490–1.364	0.440			
Ascites						
Absence	1					
Presence	0.648	0.545–1.459	0.891			
Operation History						
No	1			1		
Yes	0.691	0.425–1.125	0.138	0.928	0.556–1.548	0.775
Treatment lines of immunotherapy						
1	1			1		
>=2	1.512	0.839–2.726	0.169	1.070	0.534–2.144	0.849
CRP						
< 0.8	1					
>=0.8	1.181	0.690–2.023	0.544			
AFP						
< 20	1					
>=20	1.135	0.712–1.808	0.595			
PNI						
< 44.91	1			1		
>=44.91	0.617	0.388–0.983	0.042	0.426	0.244–0.745	0.003
Therapeutic regimen						
Anti-PD1 monotherapy	1			1		
Combination therapy	0.387	0.212–0.708	0.002	0.773	0.368–1.620	0.495
TACE simultaneously						
No	1					
Yes	0.916	0.553–1.515	0.731			
Therapeutic effect evaluation						
CR + PR	1			1		
SD + PD	3.810	2.192–6.622	< 0.001	4.104	2.211–7.618	< 0.001

At the univariate analysis for OS, Child-Pugh class, tumor size, previous surgery, CRP, PNI, therapeutic regimen, therapeutic effect evaluation substantially influenced the OS (Table 4). Multivariate analysis further revealed that previous surgery (HR = 0.463; 95%CI 0.242–0.889; P = 0.021), PNI (HR = 0.411; 95%CI 0.191–0.886; P = 0.023), therapeutic regimen (HR = 0.262; 95%CI 0.114–0.602; P = 0.002), therapeutic effect evaluation (HR = 3.287; 95%CI 1.660–6.509; P = 0.001) were the most significant factors influencing the OS.

**Table 4:** Univariate and multivariate Cox regression analyses of OS in unresectable HCC patients

	Overall Survival Univariate			Overall Survival Multivariate		
Variables	HR	95%CI	p-value	HR	95%CI	p-value
Age						
< 55 years	1					
>=55 years	0.844	0.508–1.403	0.513			
Gender						
Feale	1					
Male	1.007	0.478–2.122	0.985			
ECOG-PS						
0	1					
>=1	1.532	0.476–4.933	0.475			
Smoking history						
Never	1					
Current or former	1.120	0.673–1.864	0.664			
Drinking history						
Never	1					
Current or former	0.972	0.577–1.637	0.916			
Allergic history						
No	1					
Yes	0.640	0.274–1.491	0.301			
HBV infection						
Negative	1					
Positive	1.075	0.510–2.268	0.849			
Child-Pugh class						
A	1			1		
B	3.890	1.783–8.489	0.001	1.525	0.594–3.916	0.380
Portal vein thrombosis						
No	1					
Yes	1.277	0.735–2.217	0.385			
Extrahepatic metastasis						
No	1					
Yes	1.193	0.719–1.982	0.495			
Perihepatic lymph node metastasis						
No	1					
Yes	1.323	0.794–2.206	0.283			
BCLC score						
B	1					
C	0.993	0.561–1.913	0.983			
Tumor number						
Single	1					
Multiple	1.062	0.537-2.100	0.863			
Tumor size						
=<5	1			1		
> 5	1.669	0.984–2.831	0.058	1.049	0.511–2.152	0.897
Liver cirrhosis						
Absence	1					
Presence	0.791	0.458–1.364	0.399			
Ascites						
Absence	1					
Presence	0.763	0.443–1.315	0.330			
Previous surgery						
No	1			1		
Yes	0.440	0.263–0.737	0.002	0.463	0.242–0.889	0.021
Treatment lines of immunotherapy						
1	1					
>=2	1.399	0.742–2.635	0.299			
CRP						
< 0.8	1			1		
>=0.8	2.008	1.122–3.593	0.019	1.066	0.531–2.141	0.858
AFP						
< 20	1					
>=20	1.045	0.626–1.742	0.867			
PNI						
< 44.91	1			1		
>=44.91	0.449	0.269–0.750	0.002	0.411	0.191–0.886	0.023
Therapeutic regimen						
Anti-PD1 monotherapy	1			1		
Combination therapy	0.410	0.221–0.761	0.005	0.262	0.114–0.602	0.002
TACE simultaneously						
No	1					
Yes	0.677	0.406–1.129	0.677			
Therapeutic effect evaluation						
CR + PR	1			1		
SD + PD	2.526	1.458–4.379	0.001	3.287	1.660–6.509	0.001

These findings demonstrate that pre-treatment PNI as vital as therapeutic effect evaluation is an independent prognostic factor for predicting both PFS and OS of unresectable HCC patients treated with ICIs therapy.

## Discussion

The 2020 version of clinical oncology guidelines from the Chinese Society recommend four preferred regimens for routine first-line therapy for unresectable HCC: sorafenib, lenvatinib, donafenib, and oxaliplatin-based chemotherapy [6–[7, 25]–26]. In addition, some targeted drugs such as regorafenib and apatinib, as well as anti-PD1 monotherapy like nivolumab, pembrolizumab, and camrelizumab, have been approved for second-line treatment [11–13]. Combining immunotherapy and antiangiogenic therapy has received more extensive attention, particularly in the last two years, as a breakthrough in the treatment of advanced HCC [16]. Previous clinical trials on the effectiveness of combining anti-PD1 and antiangiogenic therapy have demonstrated ORRs ranging from 24 to 76.7% and DCRs ranging from 74 to 93.8% [16, 27–30]. The latest recommendations update the first-line treatment by adding combination immunotherapy as the preferred option [31]. In this study, we found that patients undergoing anti-PD1 combination therapy were more common in the first-line treatment for advanced HCC, whereas anti-PD1 monotherapy was more commonly used in the second line or later. Despite higher risk factors such as portal vein thrombosis being more exposed in the anti-PD1 combination therapy group (Table 1), this treatment indicated significant improvement in both short-term outcomes (ORR, DCR) and long-term outcomes (PFS, OS) compared to monotherapy (Table 1; Fig. 4). Although immunotherapy has achieved significant efficacy in unresectable HCC treatment, there is no effective potential biomarker for assessing treatment with ICIs therapy. The objective of our study was to explore effective prognostic indicators for recurrent or unresectable HCC undergoing anti-PD1 therapy.

In this study, 14 patients received ICIs monotherapy and 71 patients were in the anti-PD1 combination group. Of those, 83.5% received immunotherapy combined with antiangiogenic therapy, including 61 patients in the first-line therapy and 10 patients in the second-line therapy. Since 2017, treatment strategies for unresectable HCC have seen some remarkable developments, particularly in immunotherapy combined with angiogenesis inhibitors. Analysis of pharmacologic regimens revealed that the use of anticancer drugs was consistent with clinical trials, with the most commonly used drugs being lenvatinib, anlotinib, and apatinib (Table 5). Sorafenib was introduced earlier than other types of TKIs and played an important role in treating patients with unresectable HCC. However, sorafenib is less versatile in immunotherapy combination therapy compared to other TKIs like lenvatinib and apatinib. There could be various reasons for this, with the key one being that long-term exposure to sorafenib can increase the synthesis and secretion of hepatocyte growth factor (HGF), along with the levels of c-Met and p-Met [32]. Additionally, HGF can recruit and regulate the migration of M2 macrophages, which can increase resistance to ICIs [33]. On the other hand, lenvatinib can inhibit vascular endothelial growth factor receptor (VEGFR) and fibroblast growth factor receptor (FGFR), which may contribute to its efficacy. But more importantly, lenvatinib can modulate the tumor microenvironment (TME) and enhance the cytotoxic effect of T cells [34]. In terms of synergistically modulating effector T cells, sorafenib is inferior to lenvatinib.

**Table 5:** Pharmacologic regimens in combination group

	Types of combination drugs	Case(%)
Anti-angiogenic agent	lenvatinib	41
	sorafinib	3
	apatinib	7
	anlotinib	19
	bevacizuma	1

Anatomically, the liver is supplied with blood from two vascular systems, with about 80% of blood coming from the portal vein and 20% from the hepatic artery [35]. The portal venous drainage carries antigenic material from the mesenteric vein to the liver, and the continual exposure to intestinal system-derived antigenic material has contributed to immune tolerance in the liver. However, this physiological mechanism may also contribute to hepatocellular carcinoma’s escape from immune system surveillance. The therapeutic effect of ICIs for HCC has been shown to improve by relieving immune system depression. To some extent, the evolution of tumor immune escape overlaps with the HCC inflammatory microenvironment. Therefore, immune condition and nutritional status play a vital role in the inflammatory environment in solid tumors. Increasingly, more studies report that peripheral markers of inflammation can provide significant information in prognostic prediction. Such inflammation biomarkers could help predict the suitability and susceptibility of patients with solid tumors undergoing ICIs therapy [18]. Compared with colorectal cancer and unresectable non-small cell lung cancer, the progress of immune therapy in unresectable HCC is relatively stagnant. Our study aims to provide further evidence to support the use of immunotherapy in patients with unresectable HCC.

We determined that the optimal cut-off value for PNI was 44.91, which is consistent with the accepted value of 45 in existing literature [36]. Using this cut-off value, patients were categorized into two groups based on their pre-treatment PNI levels. We found that patients with higher pre-treatment PNI had a better prognosis compared to those with lower PNI. PNI, due to its low cost and ease of use, has the potential to serve as a biomarker to help clinicians identify advanced HCC patients with poor survival who may benefit from ICIs treatment. Univariate and multivariate Cox regression analyses in the entire population demonstrated a significant difference in PFS and OS between the PNI high group and PNI low group (as shown in Tables 3 and 4). Similar results were observed in the subgroup analysis (as shown in Fig. 4). In other words, patients with high PNI may be more responsive to anti-PD1 combination therapy or anti-PD1 monotherapy compared to those with low PNI. PNI can serve as an indirect index for assessing the curative effect of immunotherapy in unresectable HCC, as it comprises serum albumin and peripheral lymphocytes. The serum albumin fraction of plasma protein, which is mainly produced by liver cells, is not only an indicator of nutritional status but also related to liver functional reserve. As two parameters in the Child-Pugh score, serum albumin is strongly associated with ascites. The prognosis of HCC patients is widely analyzed using the BCLC staging system, which is based on the Child-Pugh score, tumor status, and performance status. Moreover, a recent study has shown a link between T cell mitochondria biogenesis and the response to anti-PD1 antibodies [37]. High levels of branched-chain amino acids (BCAAs) in the diet are associated with elevated serum albumin levels [38], and a diet rich in BCAAs has been found to improve mitochondrial biogenesis [39]. T cells with greater mitochondrial biogenesis are better equipped to fight cancer cells, which can lead to an improved overall prognosis [40]. Therefore, we can infer that diets that promote high albumin levels may enhance the response to anti-PD1 antibodies by improving mitochondrial biogenesis. Peripheral lymphocytes are closely associated with immune cells within the tumor microenvironment. The presence of immune cells, particularly lymphocyte subsets such as T-lymphocytes, B-lymphocytes, and natural killer cells, is critical for achieving a therapeutic effect. These factors may explain why PNI is a predictor of response to ICIs in patients. Therefore, improving PNI levels may enhance the prognosis of patients. Our study found that PNI is a reliable predictor of outcomes in recurrent and unresectable HCC patients receiving ICIs. The easy availability and low cost of this indicator make it a promising tool for further evaluation.

Our pilot study has several limitations that should be acknowledged. Firstly, it was conducted in a single center, which could introduce some unavoidable biases. Secondly, the relatively small sample size limited the credibility and reliability of the study, as it was restricted to an Asian population. Therefore, further confirmation within a larger clinical trial is needed. Nevertheless, the “real-world” data analyses we conducted provide valuable clues for future prospective studies. Based on the existing evidence, our study demonstrated that anti-PD1 therapy combination therapy (mainly with an antiangiogenic agent) is an effective and well-tolerated approach for unresectable HCC in the real world. This information is valuable for guiding clinical treatment. Additionally, the pretreatment PNI with an optimal cut-off value of 44.91 has the potential to be used in evaluating the prognosis of patients with unresectable HCC.

## Conclusion

Our study has highlighted that pre-treatment PNI represents a prognostic and available low-cost biomarker in recurrent or unresectable HCC patients treated with ICIs therapy, whether in anti-PD1 combination therapy or in anti-PD1 monotherapy. Considered that PNI is clinical feasible, this immune-nutritional biomarker has much to commend in clinical treatment and scientific research.

## Data Availability

All data generated or analysed during this study are included in this published article. The data of this study are available from the corresponding author on reasonable request.

## Abbreviations

AFP: Alpha fetoprotein
AUC: The area under the curve
BCAA: Branched-chain amino acids
BCLC: Barcelona Clinic Liver Cancer
CR: Complete response
DCR: Disease control rate
ECOG-PS: Eastern Cooperative Oncology Group performance status
FGFR: Fibroblast growth factor receptor
HBV: Hepatitis B virus
HCC: Hepatocellular carcinoma
HGF: Hepatocyte growth factor
ICIs: Immune checkpoint inhibitors
NLR: Neutrophil-to-lymphocyte ratio
NSAID: Nonsteroidal anti-inflammatory drug
ORR: Objective response rate
OS: Overall survival
PCR: Polymerase chain reaction
PD1: Programmed death receptor-1
PD-L1: Programmed death ligand-1
PFS: Progression-free survival
PLR: Platelet-to-lymphocyte ratio
PNI: Prognostic nutritional index
PR: Partial response
RECIST: Response evaluation criteria in solid tumors
ROC: Receiver operating characteristic curve
SD: Stable disease
SII: Systemic immune-inflammation index
TRAEs: Treatment-related adverse events
Tregs: T regulatory cells
VEGFR: Vascular endothelial growth factor receptor
